# Optimized process parameters for fabricating metal particles reinforced 5083 Al composite by friction stir processing

**DOI:** 10.1016/j.dib.2015.09.006

**Published:** 2015-09-25

**Authors:** Ranjit Bauri, Devinder Yadav, C.N. Shyam Kumar, G.D. Janaki Ram

**Affiliations:** Indian Institute of Technology Madras, Chennai 600036, India

**Keywords:** Friction stir processing, Metal matrix composites, Particle distribution

## Abstract

Metal matrix composites (MMCs) exhibit improved strength but suffer from low ductility. Metal particles reinforcement can be an alternative to retain the ductility in MMCs (Bauri and Yadav, 2010; Thakur and Gupta, 2007) [1,2]. However, processing such composites by conventional routes is difficult. The data presented here relates to friction stir processing (FSP) that was used to process metal particles reinforced aluminum matrix composites. The data is the processing parameters, rotation and traverse speeds, which were optimized to incorporate Ni particles. A wide range of parameters covering tool rotation speeds from 1000 rpm to 1800 rpm and a range of traverse speeds from 6 mm/min to 24 mm/min were explored in order to get a defect free stir zone and uniform distribution of particles. The right combination of rotation and traverse speed was found from these experiments. Both as-received coarse particles (70 μm) and ball-milled finer particles (10 μm) were incorporated in the Al matrix using the optimized parameters.

Specifications Table

**Table:** 

Subject area	*Materials Science and Engineering*
More specific subject area	*Friction stir Welding and Processing*
Type of data	*Table, images*
How data was acquired	*The tool was rotated at different rpm and traversed at different speeds at a particular rpm. Images were captured in scanning electron microscope (SEM)*
Data format	*Analyzed*
Experimental factors	The base material chosen was AA 5083 Al alloy with a nominal composition of 4.2% Mg, 0.6% Mn, 0.2% Si and 0.2% Fe. An indigenously made friction stir processing (FSP) machine was used for the FSP experiments. The tool was made of hardened M2 tool steel and had a shoulder and a pin. The tool was cylindrical with shoulder diameter of 15 mm and pin diameter and pin length of 5 mm and 3.5 mm, respectively. The tool was attached to the FSP machine and was rotated with the help of the inbuilt rotor and motor of the machine. The motor speed (rpm) and the speed of forward motion (traverse speed) of the tool were controlled through a computer using Labview software. In order to reduce the size, Ni particles were ball milled for 20 h in a planetary mill using tungsten carbide balls in toluene medium.
Experimental features	No tilt was given to the tool. A constant vertical load of 8 kN was used for all the runs (except first two). All the experiments were carried out at room temperature.
Data source location	Indian Institute of Technology Madras, Chennai, India
Data accessibility	Data is with this article

Value of the data

• The FSP parameters presented here lead to defect free stir zone and provide a processing window for incorporating Ni particles in 5083 Al to make composites by FSP.
• Similar parameters can be used to process other Al or Mg based composites with variety of reinforcement particles.
• Other researchers may use the data as guidelines to select suitable parameters without going through rigorous trials and errors.
• The data will also help in selectively enhancing the properties at desired locations on the surface offering possibility of a new kind of surface engineering.

## 1 Data

Process parameters for friction stir processing (FSP) of 5083 Al alloy for a defect free processed zone (stir zone) and for incorporating metal particles to make composites. The parameters are the rotation speed (rpm) and the traverse speed (speed of forward motion of the tool). The particle size and the micrographs depicting the structure of the composite are the other parts of the data presented here.

## 2 Experimental design, materials and methods

**Fig. 1:**
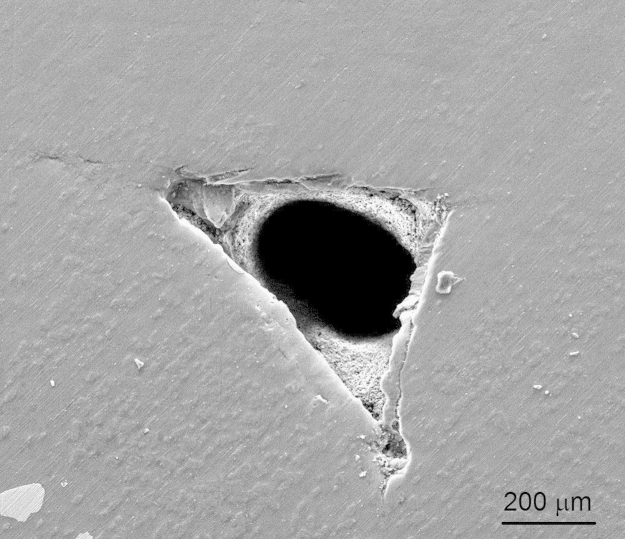
SEM micrograph showing tunnel defect.

**Fig. 2:**
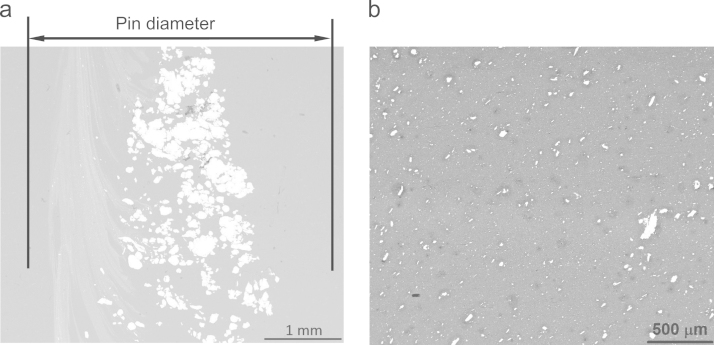
SEM micrographs showing particle distribution of (a) coarse (70 μm) and (b) ball milled finer (10 μm) particles. Rotation speed 1200 rpm and traverse speed 24 mm/min.

A friction stir processing setup, which was obtained by modifying a milling machine, was used. In order to incorporate the particles, a groove of 1 mm in width and 2 mm in depth was cut precisely on the 5083 Al plate. The groove was first filled with Ni particles of average size 70 μm and FSP was carried out over the groove with a cylindrical tool. More details can be found in the parent article [3]. The first objective of FSP should be to obtain a stir zone free of tunnel defect (Fig. 1) or any other kind of defects and the process parameters should be optimized accordingly. In order to make a composite by FSP, the incorporation and proper distribution of the particles also had to be taken care while optimizing the parameters. As shown in Table 1, several process parameters with many combinations of rotation and traverse speeds were explored to get a defect free stir zone and incorporate the particles uniformly. A rotational speed of 1200 rpm and traverse speed of 24 mm/min gave rise to a defect free stir zone in the friction stir processed base alloy and the composite processed by incorporating Ni particles. However, the dispersion of the coarse particles was not uniform as shown in Fig. 2(a) and many of the particles also got fractured consuming much of the input energy.

The heat input into the material during FSP depends on the ratio of rotation to traverse speeds with higher ratio giving rise to higher heat input [4,5]. Therefore, the traverse speed was reduced to15 and 12 mm/min (Table 1, experiment # 16, 17) to produce more heat input into the material. This however, did not improve the dispersion of the coarse Ni particles. Even a higher rotation speed of 1500 rpm (experiment 18) also did not help in this regard. As shown in Fig. 2(a), the particles were largely dispersed around the pin diameter and rest of the stir zone was virtually free of particles. The particle size was then refined to 10 μm by ball milling. As shown in Fig. 2(b) the ball milled finer particles were dispersed uniformly throughout the stir zone.

## Figures and Tables

**Table 1:** FSP Process parameters optimization.

Expt. No.	Rotation speed (rpm)	Traverse speed (mm/min)	Vertical load (kN)	Observation/comments
1.	1000	12	5	Defect – tunnel hole in the nugget
2.	1200	12	5	Tunnel hole
3.	1000	12	8	Tunnel hole
4.	1200	12	8	Smaller tunnel hole
5.	1500	12	8	Tunnel hole
6.	1800	12	8	Tunnel hole
7.	1000	18	8	Tunnel hole
8.	1500	18	8	Tunnel hole
9.	1800	18	8	Tunnel hole
10.	1500	15	8	Tunnel hole
11.	1200	6	8	Tunnel hole
12.	1500	6	8	Tunnel hole
13.	1200	30	8	Tunnel hole
14.	1200	24	8	NO defect
15.	1200	24	8	Coarse Ni particles added. Particle clustering
16.	1200	15	8	Defect, particle clustering
17.	1200	12	8	Defect, particle clustering
18.	1500	12	8	Particle clustering
19.	1500	6	8	Particle clustering
20.	1200	24	8	Ball milled finer particles. Uniform distribution
